# Percutaneous transforaminal endoscopic decompression with removal of the posterosuperior region underneath the slipping vertebral body for lumbar spinal stenosis with degenerative lumbar spondylolisthesis: a retrospective study

**DOI:** 10.1186/s12891-024-07267-7

**Published:** 2024-02-20

**Authors:** Rongbo Yu, Xiaokang Cheng, Bin Chen

**Affiliations:** 1 Department of Minimally Invasive Spine Surgery, Chengde Medical University Affiliated Hospital, Chengde, 067000 Hebei China; 2 Department of Orthopedic, Beijing Tongren Hospital Affiliated to Capital Medical University, Beijing, 100730 China

**Keywords:** Percutaneous transforaminal endoscopic decompression, Lumbar spinal stenosis, Degenerative lumbar spondylolisthesis, Posterosuperior region underneath the slipping vertebral body, Bone drill, Local anesthesia

## Abstract

**Background:**

Percutaneous transforaminal endoscopic decompression (PTED) is an ideal minimally invasive decompression technique for the treatment of lumbar spinal stenosis (LSS) with degenerative lumbar spondylolisthesis (DLS). The posterosuperior region underneath the slipping vertebral body (PRSVB) formed by DLS is an important factor exacerbating LSS in patients. Therefore, the necessity of removing the PRSVB during ventral decompression remains to be discussed. This study aimed to describe the procedure of PTED combined with the removal of the PRSVB and to evaluate the clinical outcomes.

**Methods:**

LSS with DLS was diagnosed in 44 consecutive patients at our institution from January 2019 to July 2021, and they underwent PTED combined with the removal of the PRSVB. All patients were followed up for at least 12 months. The clinical outcomes were evaluated using the visual analog scale (VAS), Oswestry Disability Index (ODI), and modified MacNab criteria.

**Results:**

The mean age of the patients was 69.5 ± 7.1 years. The mean preoperative ODI score, VAS score of the low back, and VAS score of the leg were 68.3 ± 10.8, 5.8 ± 1.0, and 7.7 ± 1.1, respectively, which improved to 18.8 ± 5.0, 1.4 ± 0.8, and 1.6 ± 0.7, respectively, at 12 months postoperatively. The proportion of patients presenting “good” and “excellent” ratings according to the modified MacNab criteria was 93.2%. The percent slippage in spondylolisthesis preoperatively (16.0% ± 3.3%) and at the end of follow-up (15.8% ± 3.3%) did not differ significantly (*p*>0.05). One patient had a dural tear, and one patient had postoperative dysesthesia.

**Conclusions:**

Increasing the removal of PRSVB during the PTED process may be a beneficial surgical procedure for alleviating clinical symptoms in patients with LSS and DLS. However, long-term follow-up is needed to study clinical effects.

**Supplementary Information:**

The online version contains supplementary material available at 10.1186/s12891-024-07267-7.

## Background

Degenerative lumbar spondylolisthesis (DLS) is a common clinical degenerative disease that often affects the health of middle-aged and older adults [1]. The open method of decompression with instrument fusion is considered the gold standard surgery for treating DLS. However, the population with DLS often comprises elderly patients with complex diseases, who may be contraindicated for surgery due to the inability to tolerate general anesthesia. Moreover, there could be a risk of significant trauma, prolonged surgical time, secondary instability [2, 3], or degeneration of adjacent segments [4]. Therefore, a simplified spinal surgical plan is needed for such patients.

Recently, percutaneous spinal endoscopy has achieved satisfactory clinical results in lumbar degenerative diseases, of which percutaneous transforaminal endoscopic decompression (PTED) is an ultra-minimally invasive surgical technique [5]. Compared with traditional decompression, PTED has the advantages of local anesthesia use, small incision size, less tissue damage, less blood loss, and rapid recovery [6]. Furthermore, it has the important advantage of retaining the biomechanical structure of the affected segment [7]. Natural processes indicate that elderly patients with DLS can achieve spontaneous fusion and reach a stable stage [8]; hence, the possibility of PTED affecting the natural process of DLS is minimal, and it has limited effects on lumbar instability.

When LSS occurs with DLS, the sliding between the vertebral bodies causes the posterosuperior region underneath the slipping vertebral body (PRSVB) to invade the lumbar spinal canal and form a stepped shape [9]. This is a pathological factor exacerbating spinal stenosis and compressing the nerve roots and cauda equina [10]. To achieve comprehensive and complete decompression, it is necessary to ensure the ventral and dorsal decompression of the nerves. By resecting the PRSVB on the ventral side, it could be possible to achieve decompression of the nerves and cauda equina. Therefore, further research on this topic is warranted to perform PTED for the treatment of LSS with DLS. Only a few studies specifically report the details of removing the PRSVB. Therefore, this study aimed to describe the procedure of PTED with the removal of the PRSVB for the treatment of LSS with DLS and to analyze its clinical results.

## Methods

### Patient population

PTED with the removal of the PRSVB was performed in 44 consecutive patients with single-level LSS and DLS. The inclusion criteria were as follows: (1) Confirmed by clinical manifestations, physical examination, and imaging as low-grade DLS (Meyerding Grade I–II) with LSS (Schizas A-C grade) [11]; (2) neurogenic claudication with unilateral or bilateral leg symptoms; and (3) non-responsive to conservative therapies for > 3 months. The exclusion criteria were as follows: (1) main low back pain symptoms; (2) isthmic lumbar spondylolisthesis confirmed on imaging; (3) segmental instability before operation (> 3 mm motion on the flexion-extension radiograph of standing position or angular displacement > 10°) [12, 13]; (4) previous history of lumbar surgery; and (5) pathologic conditions of the lumbar spine, such as trauma, tumor.

### Surgical techniques

All patients underwent PTED procedure under local anesthesia. The patient was made to lie in the lateral decubitus position, and a lumbar pad was placed under the waist to open the intervertebral disc space. The skin target puncture point was located at the symptom side and 8 − 12 cm laterally from the midline. The operation steps were carried out according to a previous study [14]. The surgical procedure consisted of three steps:1) foraminoplasty; 2) discectomy; and 3) removing the PRSVB.

**Fig. 1:**
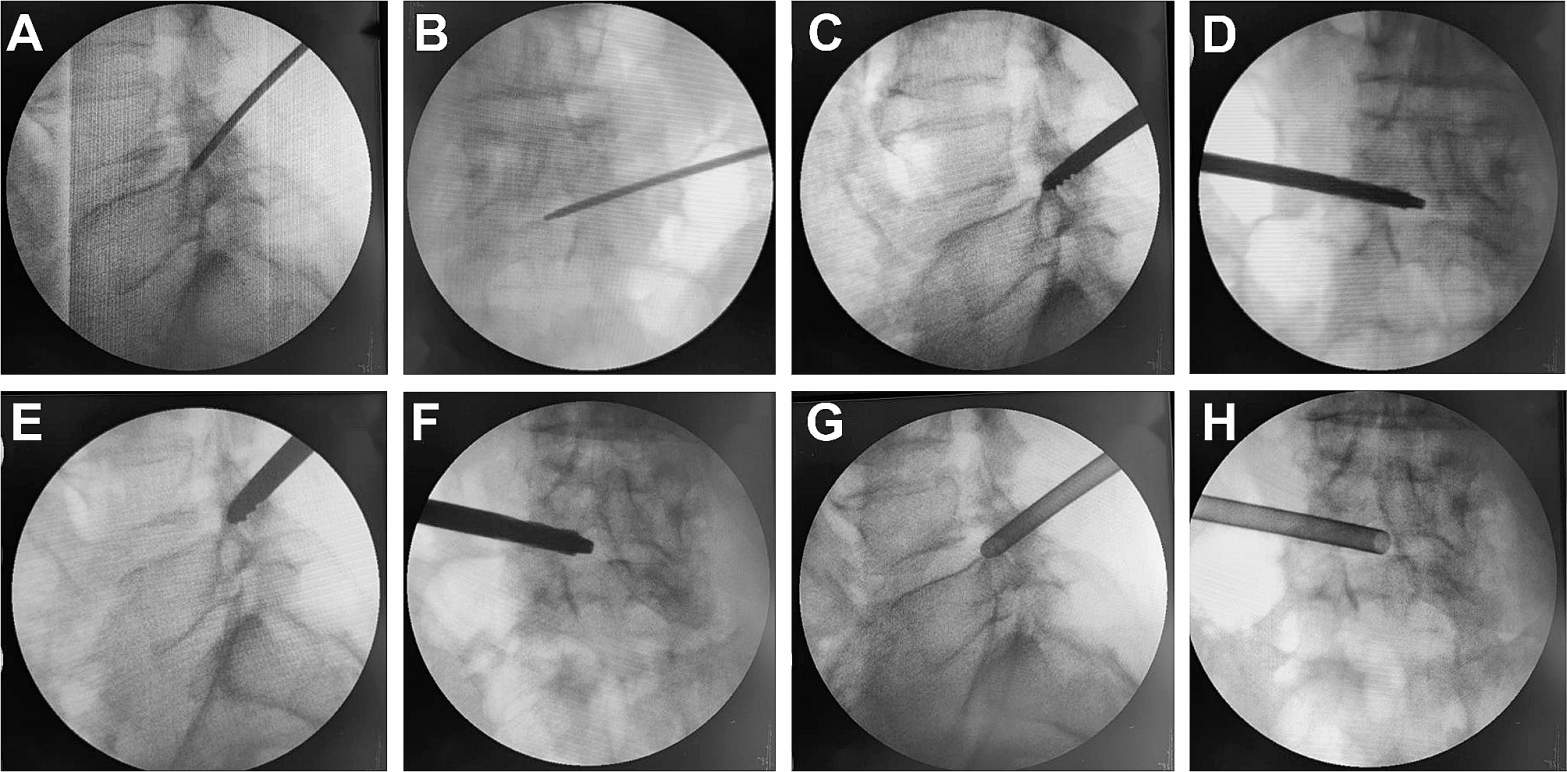
Intraoperative perspective images for establishing work channels. Sagittal (**A**) and anteroposterior (**B**) fluoroscopic images of the Tom Shidi. 6 mm bone drill (**C** and **D**) and 8 mm bone drill (**E** and **F**) were used to remove the soft and osseous tissues. Sagittal (**G**) and anteroposterior (**H**) fluoroscopic images of the working cannula

**Fig. 2:**
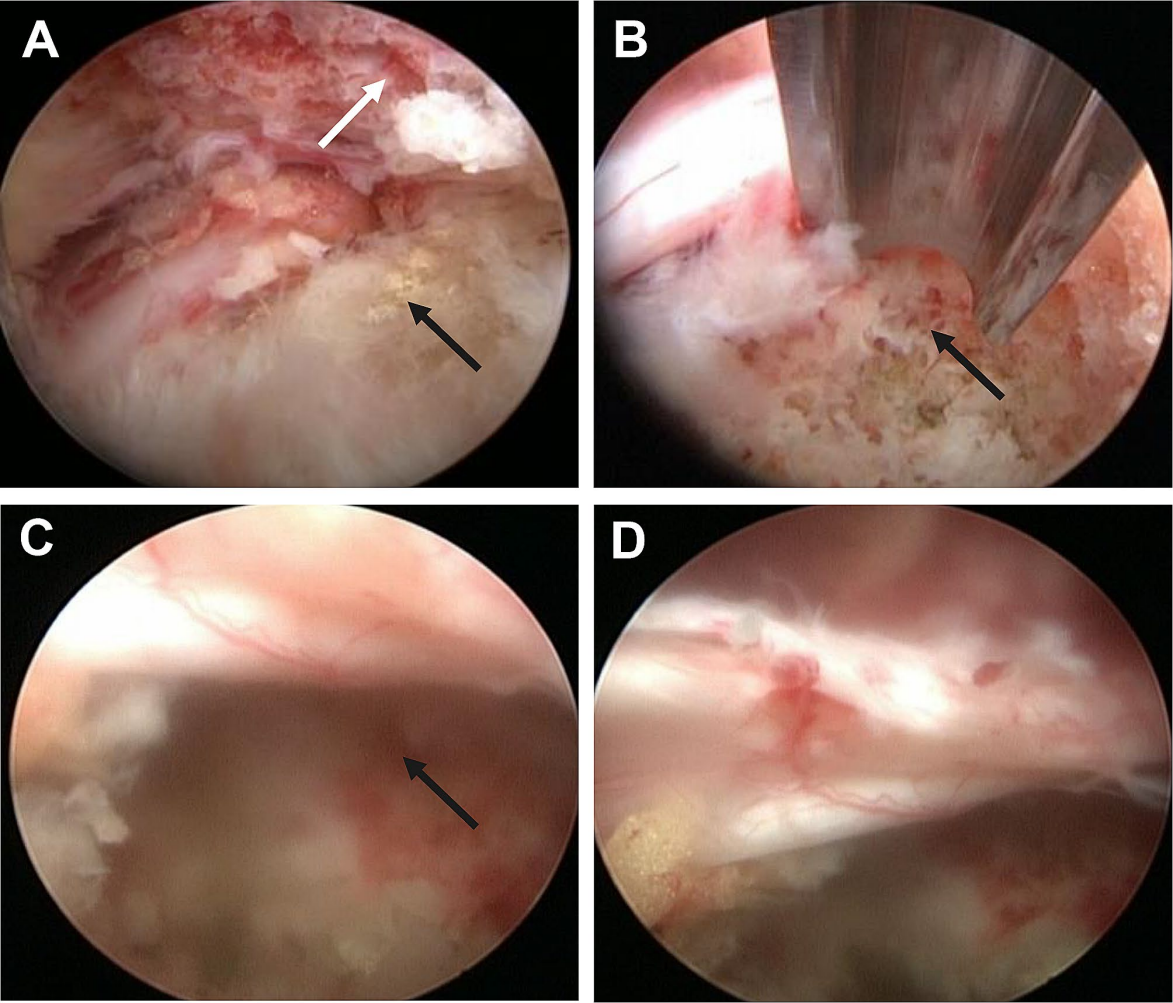
Intraoperative endoscopic views. (**A** and **B**) The PRSVB was removed with an endoscopic bone knife. (**C** and **D**) Dorsal and ventral L5 nerve roots were fully decompressed. The black arrow represents the PRSVB. The white arrow represents the superior articular process of L5

We used 3–5 mL of 1% lidocaine to penetrate the skin and then anesthetized the puncture route, foramen area and small joints with 20–25 mL of 1% lidocaine using an 18-G needle under fluoroscopy guidance. Then, a guide wire was passed through the needle and a skin incision (0.8 cm) was formed at the entrance of the guide wire. According to the direction of the guide wire, the TomShidi needle was inserted and installed, penetrating the ventral part of the superior articular process (SAP) (Fig. 1A and B). Sequential bone drills (6, 7, and 8 mm; MaxMore spine, Hoogland Spine Products GmbH, Germany) were used along the guide wire. If the surgeon was proficient in surgery, the 7-mm bone drill was not used. When the end of the bone drill gradually approached the posterior midline, it meant that the resection of ligamentum flavum (LF) and the hyperplasia osteophyte on the ventral side of the L5 SAP was completed (Fig. 1C–F). A working cannula was installed along the guide wire (Fig. 1G and H), and the prepared endoscope system was inserted into the working channel for decompression. A microscopic bone knife was used to remove the bone blocking the visual field, such as the SAP and inferior articular processes, which released the dorsal space of the nerve root. The ipsilateral LF, hypertrophic posterior longitudinal ligament, extruded disc material, and perineural scar were removed using different nucleus forceps. Following this, the PRSVB was exposed, and the key of the PTED was to remove the PRSVB using an endoscopic bone knife for full ventral decompression (Fig. 2A and B). It was necessary to perform the removal gradually to prevent bleeding and disruption of the surgery. The direction of the working cannula was adjusted to decompress the proximal end of the nerve root and nerve root canal, and the intradiscal and periannular fragments were finally confirmed to be completely removed. Bipolar radiofrequency was used to shrink the annular tears. In patients with central spinal stenosis (CSS), the position of the working cannula had to be changed after ipsilateral decompression for contralateral decompression. At this time, the nerve roots after complete decompression were not only fully exposed but also kept beating freely with the heart rate (Fig. 2C and D). Routine exploration of the exiting root was conducted. The video of endoscopic surgery can be found in additional file 1.

**Fig. 3:**
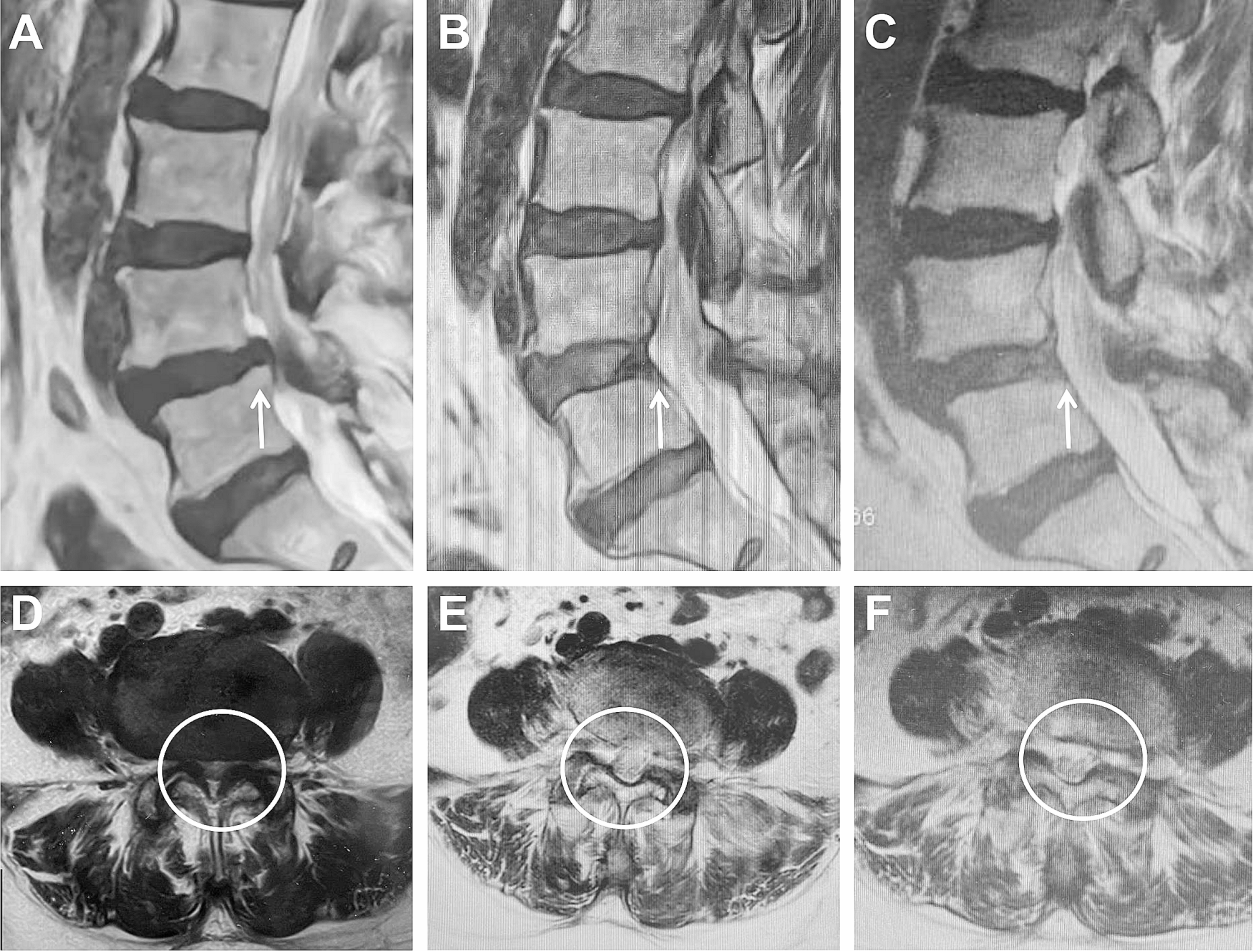
Pre- and postoperative MRI. (**A**) Preoperative MRI showed that DLS caused a posterosuperior protruded compression underneath the slipping vertebral body in the lumbar spinal canal, forming a stepped shape (white arrow). (**D**) The central spinal canal stenosis, foraminal stenosis and lateral recess stenosis (white circle). (**B, E**) On the third day after operation, the MRI revealed the PRSVB were fully removed (white arrow), and lateral recess, foramen and the central spinal canal were enlarged (white circle). (**C, F**) The MRI examination showed no evidence of significant further compression at the twelve months after surgery

**Fig. 4:**
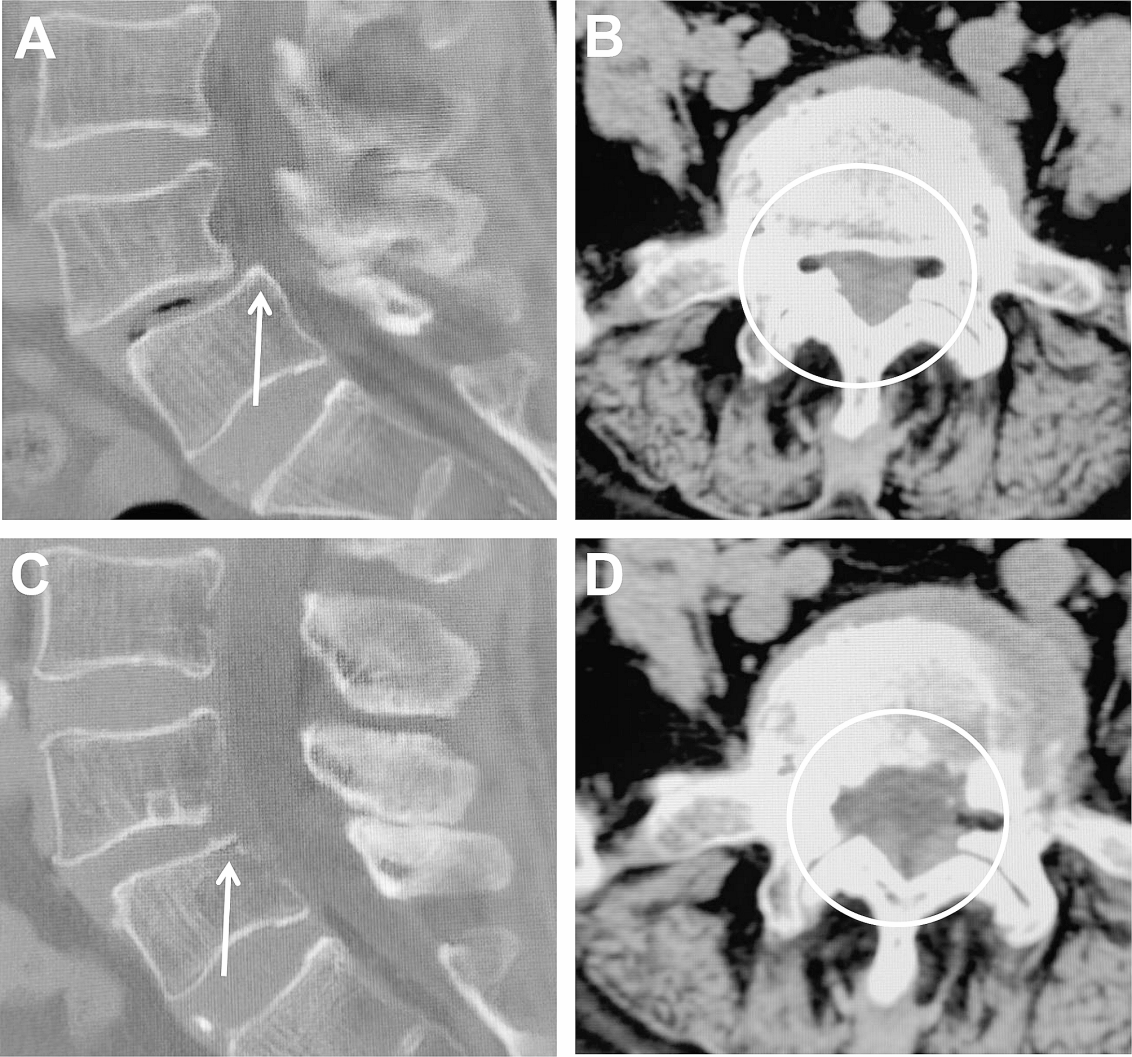
Pre- and postoperative CT. (**A, B**) Preoperative CT showed that the PRSVB (white arrow), the central spinal canal stenosis, foraminal stenosis and lateral recess stenosis (white circle). (**C, D**) On the third day after operation, the CT showed complete decompression of the nerves and removal of PRSVB (white arrow). The ventral and dorsal edges of the spinal canal became smooth and continuous. The lateral recess, foramen, and central spinal canal were enlarged (white circle)

After careful hemostasis, a drainage tube was installed along the working cannula. All patients underwent postoperative magnetic resonance imaging (MRI) or computed tomography (CT) 3 days postoperatively (Figs. 3 and 4).

### Clinical assessments

The visual analog scale (VAS), Oswestry Disability Index (ODI), and modified MacNab criteria were used to evaluate the clinical outcomes [15]. The operation duration, postoperative hospital stay, estimated blood loss and drainage volume were recorded. All patients routinely underwent CT and MRI after 3 days to confirm complete decompression. At 12 months postoperatively, all patients underwent MRI or CT to confirm no recurrence of LSS with DLS. The percentage of slip before and after operation was evaluated by using standing lumbar lateral radiography. The percentage slippage was defined as the ratio of relative displacement distance between the involved vertebrae and the horizontal length of the slipped vertebral body [16, 17].

### Statistical analysis

The clinical results were analyzed statistically using SPSS 26.0 (IBM, Armonk, USA). To compare the mean outcome scores from pre- and postoperative variables, paired t tests or Wilcoxon rank sum test were used. *P* < 0.05 was considered statistically significant.

## Results

### Preoperative demographic characteristics

Table 1 shows the preoperative demographic characteristics. The study sample included 13 men and 31 women. The average follow-up period was at least 12 months (range, 12–24 months). All cases were classified as Meyerding Grade I of DLS, and imaging evaluation revealed that spondylolisthesis was located at L4 − 5 in all the patients. The average age of the patients was 69.5 years (range, 60–83 years), and the average duration of symptoms was 40.1 months (range, 4–120 months). The most common comorbidity was cardiovascular diseases (40.9%), such as hypertensive disorders (Table 2), followed by endocrinology diseases (22.7%). Only seven patients had no comorbidities.

**Table 1:** Demographics of included patients

	Value (Mean ± SD)
Sex (M/F, n)	13/31
age (years)	69.5 ± 6.9
Duration of symptoms(months)	40.1 ± 33.5
L4 − 5	44
Blood loss(mL)	13.9 ± 5.6
Drainage (mL)	27.8 ± 13.0
Duration of operation (minutes)	67.8 ± 15.9
Postoperative hospital stay (days)	4.2 ± 1.4

**Table 2:** Comorbidities

	Number of Patients	Percentage
Cardiovascular	18/44	40.9%
Endocrinologic	10/44	22.7%
Pulmonary	5/44	11.4%
Hepatobiliary	4/44	9.0%
Urologic	4/44	9.0%
Cerebrovascular	3/44	7.0%
Others	5/44	11.4%

### Clinical results

**Fig. 5:**
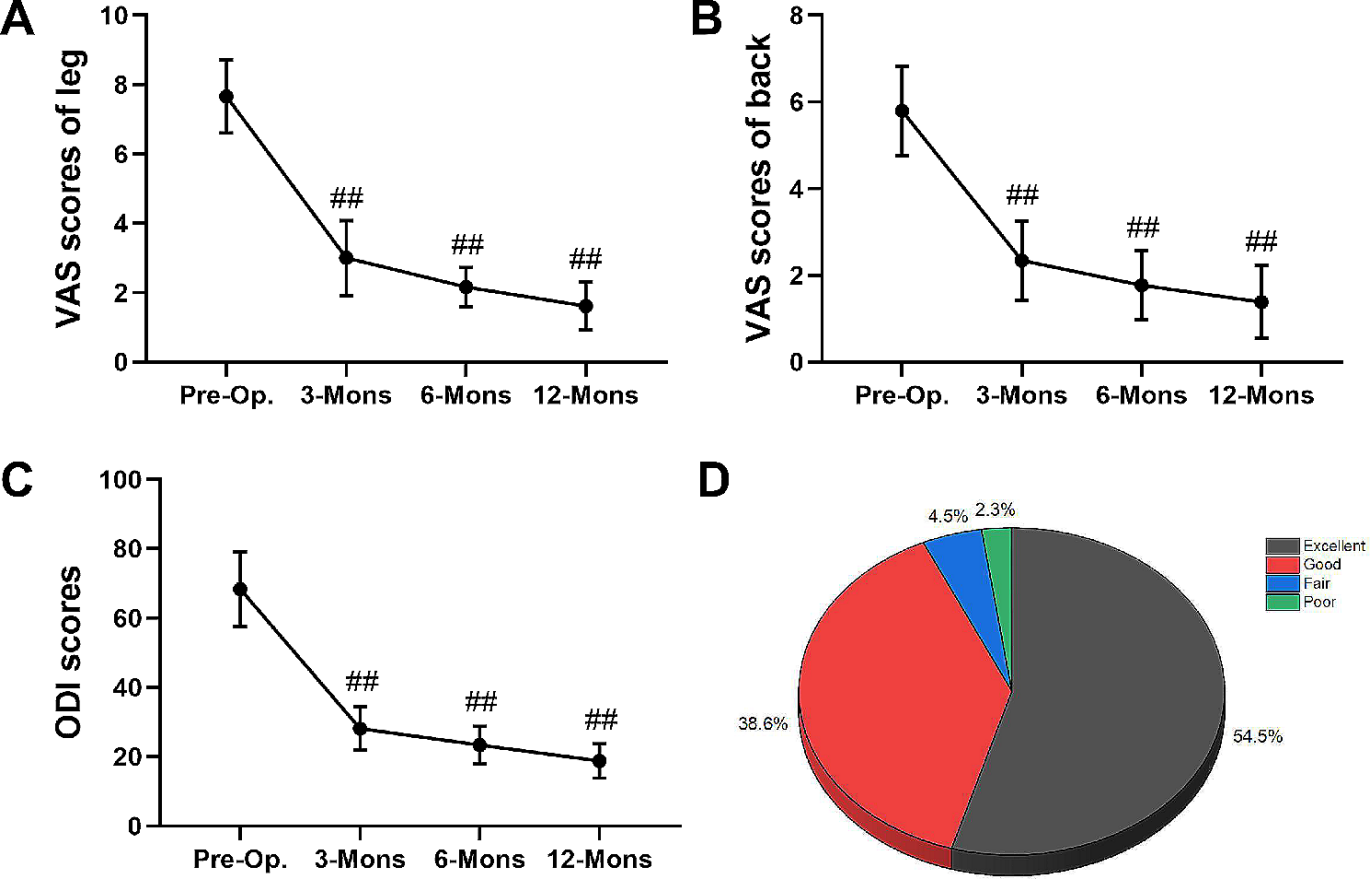
Clinical outcomes before and after PTED at different follow-up time points. (**A**) VAS scores for low back pain. (**B**) VAS scores for leg pain. (**C**) ODI scores before and after PTED. (**D**) Outcome of the modified MacNab criteria. # #*P* < 0.01 vs. pre‑operation group

**Fig. 6:**
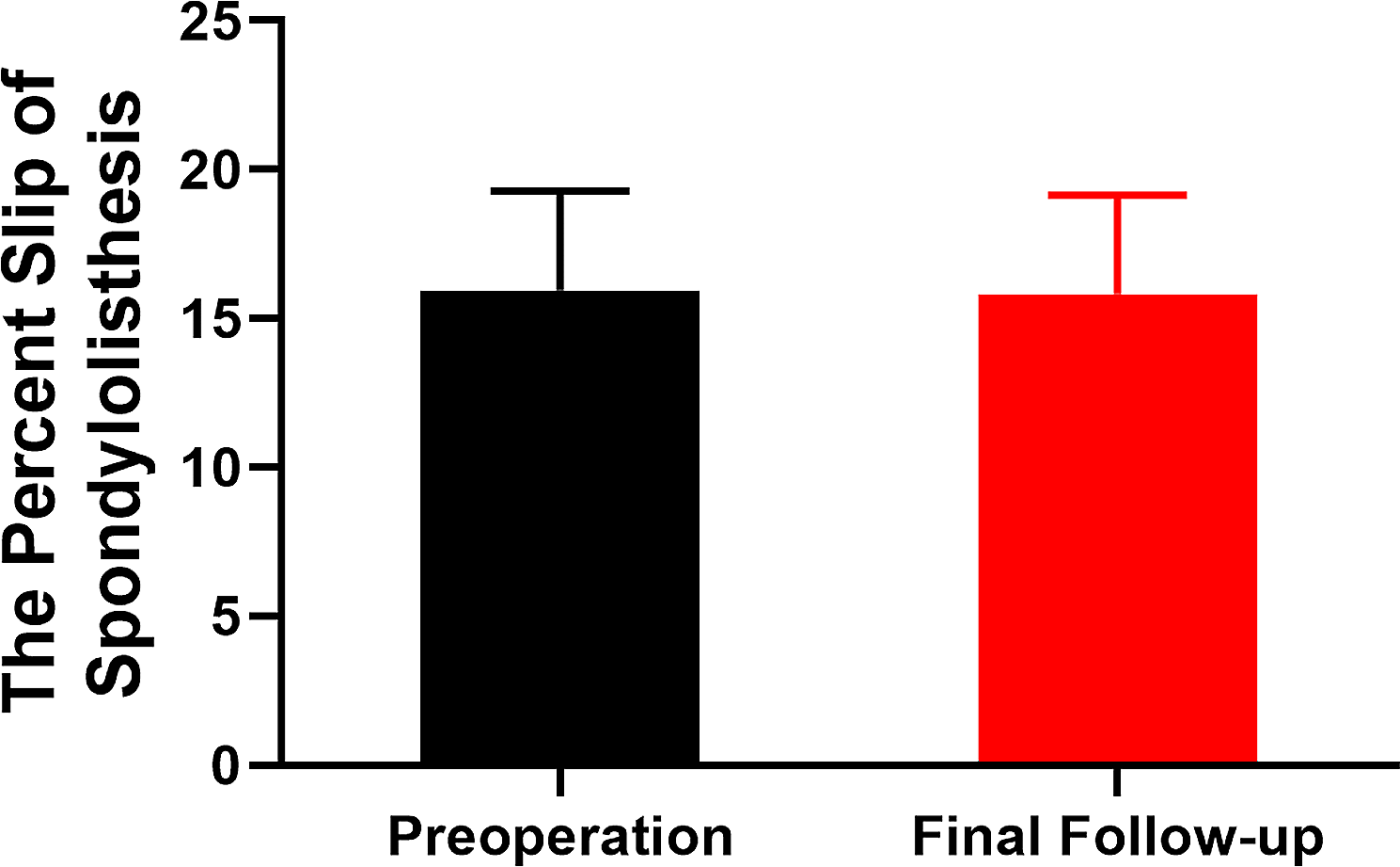
Percent slip of spondylolisthesis before surgery and at final follow-up was compared. No statistically significant differences were observed between them (*P* > 0.05)

The average operation time, postoperative hospital stay, blood loss, and drainage volume were 67.8 min (range, 44 − 105 min), 4.2 days (range, 2–8 days), 13.9 mL (range, 5–25 mL), and 27.8 mL (range, 10–60 mL), respectively. The mean preoperative ODI score, VAS score of the low back, and VAS score of the leg were 68.3 ± 10.8, 5.8 ± 1.0, and 7.7 ± 1.1, respectively, which improved to 18.8 ± 5.0, 1.4 ± 0.8, and 1.6 ± 0.7, respectively, at 12 months postoperatively (Table 3). The postoperative VAS and ODI scores at 3, 6, and 12 months were significantly lower than the preoperative scores (*P* < 0.01) (Fig. 5A–C). The modified MacNab criteria was applied to every patient, the results were as follows: excellent in 24 patients (54.5%), good in 17 (38.6%), fair in 2 (4.5%), and poor in 1 (2.3%) (Fig. 5D). The average percentage of slippage in spondylolisthesis was 16.0% ± 3.3% preoperatively and 15.8% ± 3.3% during the final follow-up (Fig. 6), with no statistically significant differences between them (*P* > 0.05). MRI showed no evidence of significant further compression in any patient at 12 months postoperatively (Fig. 3C and F).

**Table 3:** Clinical outcomes before and after endoscopic decompression at different follow-up time points

Time Point	Pre-Op.	3-Mons	6-Mons	12-Mons
VAS of leg pain	7.7 ± 1.1	3.0 ± 1.1**	2.2 ± 0.6**	1.6 ± 0.7**
VAS of back pain	5.8 ± 1.0	2.3 ± 0.9**	1.8 ± 0.8**	1.4 ± 0.8**
ODI (%)	68.3 ± 10.8	28.1 ± 6.3**	23.3 ± 5.4**	18.8 ± 5.0**

Note: **P < 0.01 versus Pre-Op.

### Complications

One patient experienced a very small dural tear, which was not sutured during the operation; however, no permanent neurological sequelae were reported during follow-up. One patient had postoperative dysesthesia, and the pain was relieved after an epidural injection of glucocorticoids plus lidocaine. Furthermore, one patient had residue of herniation and underwent revision surgery after failure of conservative treatment. During the final follow‑up, the symptoms of the patient were relieved, and no recurrence was reported in the subsequent follow-up.

## Discussion

In patients with LSS with DLS, most experts currently believe that conservative treatment failure for at least 3–6 months is an indication for surgery [18]. Two surgical methods are commonly used nowadays, namely decompression alone and decompression combined with fusion. It is widely recognized that patients with unstable DLS, especially those with obvious axial pain, will benefit from fusion [19]. Several studies have shown that there are two criteria for selecting decompression alone in a population of patients with DLS and LSS: (1) mainly characterized by symptoms of radicular pain and neurogenic claudication [20]; (2) stable DLS (sagittal translation ≤ 3 mm on standing extension-flexion radiographs radiograph or angular displacement ≤ 10°) [12, 13]. Therefore, for patients with stable DLS and LSS with neurological symptom, the main purpose of surgery is to relieve nerve compression, and vertebral fusion and fixation are not necessary. Decompression without fusion has many advantages, including reduced complexity of surgery, short hospitalization time, and reduced cost [21]. Moreover, because patients with LSS and DLS are usually old and weak and have comorbidities, the open instrument fusion decompression method performed under general anesthesia is associated with high perioperative risks. Therefore, it is important to consider the necessity of instrument fusion in elderly patients with comorbidities. Recently, with the development of spinal endoscopy, PTED has been considered an alternative to fusion surgery due to its cost-effectiveness, low complications, reduced surgical time and blood loss [22], and minimal impact on spinal stability [14]. A summary of current state of research on transforaminal approaches for DLS is presented in Table 4.

**Table 4:** A summary of current literatures regarding transforaminal approaches to degenerative lumbar spondylolisthesis

Author	Year		Number of cases	Outcome	Follow up time	Remark
Lee et al.	2004	3		The mean ODI score improved from 77.0–24.5%.	10.7(6–15) months	DLS with compressive central canal stenosis
Jasper et al.	2014	1		One week, six months, and twelve months after the surgery the patient no longer experienced pain in his right leg. The right foot drop was significantly improved.	12 months	DLS at L5-S1 with a right neural foraminal disc herniation and osteophytic ridge complex
Jasper et al.	2014	21		Macnab showed good‑to‑excellent rate was 81.0%. The mean preoperative Vas was 8.5, which improved to 2.3 at 1 year postoperatively.	12 months	DLS
Krishnan et al.	2019	1		ODI improved from 95.6 to 8.0 at three weeks, and maintained at 39 months of follow-up. VAS (leg) improved from 9 left/6 right to 0/0 bilateral, respectively.	39 months	DLS with lumbar disc herniation
Li et al.	2019	18		Macnab showed good‑to‑excellent rate was 83.3%. The mean preoperative ODI was 68.2 ± 6.5, which improved to 31.7 ± 5.2 at the final follow up.	27.7 (24‑33) months	DLS with LSS in elderly patients
Li et al.	2019	26		Macnab showed good‑to‑excellent rate was 81.3%. The mean preoperative ODI was 64.7 ± 8.1, which improved to 31.4 ± 5.6 at the final follow up.	26.3 (24–33) months	DLS with LSS
Cheng et al.	2020	40		Macnab showed good‑to‑excellent rate was 87.5%. The mean preoperative ODI was 67.3 ± 9.3, which improved to 20.7 ± 8.1 at 1 year postoperatively.	at least 1 year	DLS with LSS in elderly patients
Cheng et al.	2020	30		Macnab showed good‑to‑excellent rate was 93.3%. The mean preoperative ODI was 67.2 ± 8.4, which improved to 19.9 ± 8.1 at 1 year postoperatively.	at least 1 year	Geriatric patients with central spinal stenosis and DLS
Wu et al.	2021	24		Macnab showed good‑to‑excellent rate was 87.5%. The mean preoperative ODI was 55.4 ± 4.4, which improved to 21.1 ± 4.4 at the final follow up.	at least 5 years	DLS
Ahn et al.	2022	22		Macnab showed good‑to‑excellent rate was 90.9%. The mean preoperative ODI was 74.8 ± 8.3, which improved to 18.18 ± 7.7 at 1 year postoperatively.	12 months	Foraminal stenosis in DLS
Ahn et al.	2022	23		Macnab showed good‑to‑excellent rate was 90.5%. The mean preoperative ODI was 75.1 ± 8.4, which improved to 18.2 ± 7.5 at 2 years postoperatively.	2 years	Foraminal stenosis in DLS

DLS and LSS can elicit three different types of pain through different mechanisms [23]. The first type is mechanical lower back pain, and the main cause of this pathology is the degeneration of the intervertebral discs and facet joints or lumbar spine instability. The second type is neurogenic claudication, which is caused by concomitant CSS secondary to DLS as well as hypertrophy of the LF and osteophytes due to facet arthrosis invading the spinal canal. The third type is pain due to compression of the nerve roots in the lateral recess or foramen caused by DLS. We explored the second and third types of pain in this study, and the main symptoms of the patients were neurogenic claudication or root pain caused by LSS combined with low-level stable DLS. Therefore, the main goal of surgery was to alleviate nerve root symptoms and neurogenic claudication and to decompress LSS exacerbated by DLS. We introduced the procedure of removing the PRSVB during PTED, which can expand the lumbar spinal canal without excessive small joint resection. We observed significant improvements in the VAS and ODI scores at 3 months, 6 months, and 12 months postoperatively. According to the modified MacNab criteria, the “excellent” and “good” rating was reported in approximately 93.2% of patients, which was better than that reported with other endoscopic treatments [24, 25]. Furthermore, the incidence of surgery-related complications (6.8%) in this study was not significantly higher than that in previous endoscopic studies [16, 26]. And 12 months after surgery, all patients underwent MRI to confirm no recurrence. Thus, it was preliminarily indicated that PTED combined with removing the PRSVB elicited a good clinical effect in patients with DLS and LSS.

The PRSVB caused by DLS can be combined into three pathological features based on the basic pathological conditions of LSS. The first is the most common case related to lateral recess stenosis [16], which is usually caused by disc herniation, hypertrophy of the LF and articular processes. The uneven PRSVB may combine with these factors to exacerbate the compression of nerve root. The second case is related to CSS. Hypertrophic articular processes and LF and hyperplastic osteophytes often invade the spinal canal in patients with severe LSS The uneven PRSVB that protrudes into the spinal canal further reduces the space of the spinal canal, leading to the occurrence of CSS and aggravating claudication [14]. The reduction in effective activity space of the cauda equina nerve and traversing nerve roots exacerbates claudication. The third case is related to intervertebral foramen stenosis. In patients with advanced DLS, PRSVB is the main factor causing intervertebral foramen stenosis due to anatomical variation and the decrease of intervertebral foramen height [26]. Thus, any factor that causes LSS may combine with PRSVB formed by DLS, and the aforementioned pathological conditions can exist alone or in combination to worsen the symptoms of the patient.

**Fig. 7:**
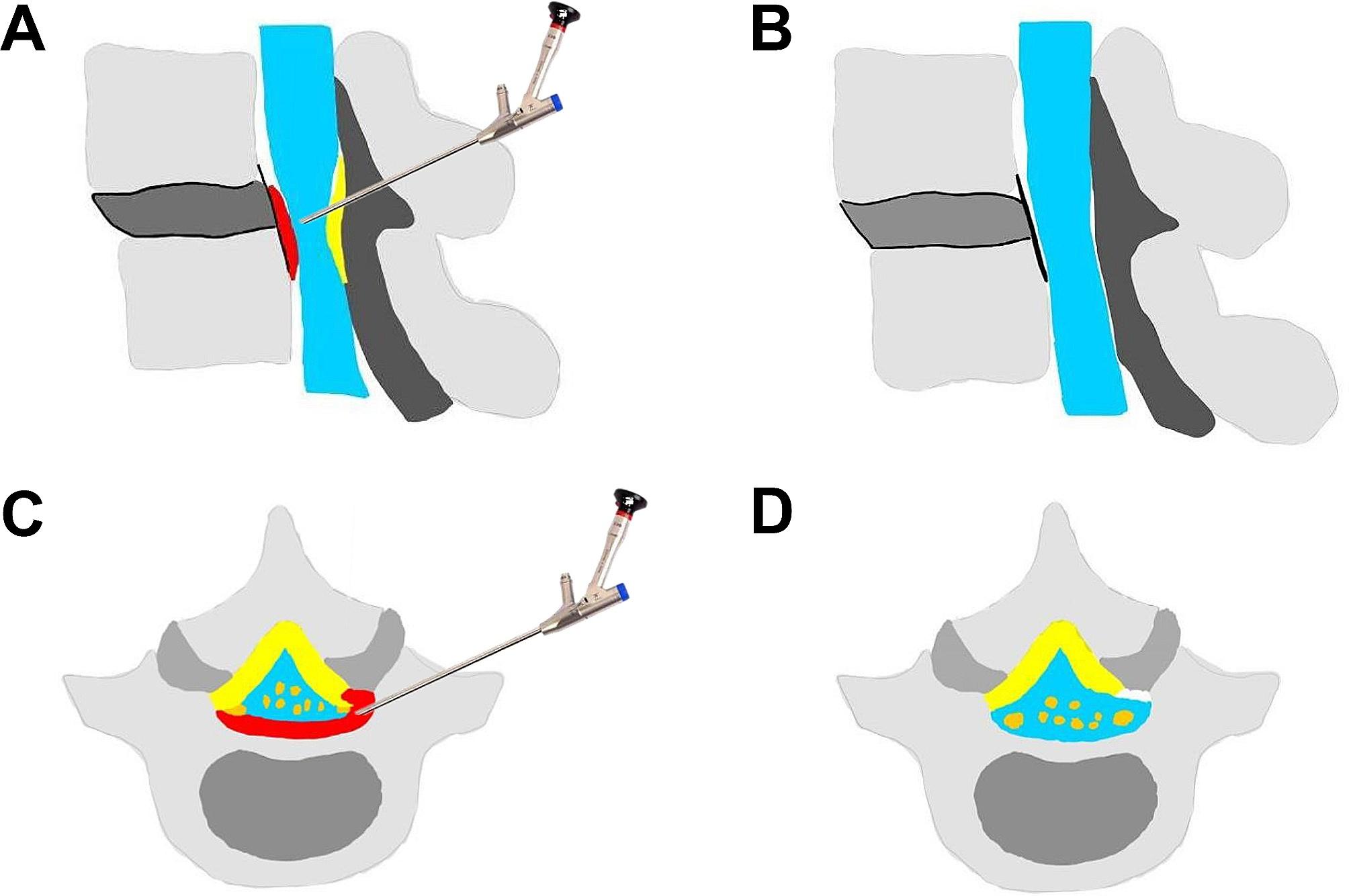
Illustrations of the endoscopic decompression procedure. (**A, C**) The special pathological compression of DLS with LSS was shown on the sagittal (**A**) and cross-sectional planes (**C**), (**B, D**) The PRSVB and hypertrophic LF were removed under endoscopy. The final view of the state of nerve decompression and the enlargement of spinal canal, foramen and lateral recess

Jasper et al. [25] reported that among the 21 patients with DLS who had undergone PTED, 3 (11.9%) relapsed in the first 3 months. The relapsed patients actively underwent enlargement of the foramen and resection of the PRSVB during revision surgery, and they recovered well postoperatively. This indicated that to achieve comprehensive decompression, attention must be paid to the removal of the PRSVB. To make decompression efficient and thorough on the dorsal and ventral sides, we used bone drills in the first foraminoplasty, which enlarge the foramen and remove the LF efficiently and safely [24]. Notably, the presence of PRSVB reduces the effective space in the spinal canal, causing the nerve roots and dura mater to be squeezed and deformed. The position of the bone drill should not blindly pursue the midline of the spine, as it could easily damage the dura mater and nerve roots. When the patient feels pain, the orthopedic surgeon should withdraw the bone drill in time and insert the working channel directly to perform foraminoplasty endoscopically. We introduced a microscopic bone knife during the second foraminoplasty, which can remove the bony structure blocking the field of vision as needed, and more importantly, it can flexibly remove the PRSVB. After 270° decompression of the ipsilateral nerve root, the abduction angle of the endoscope needs to be increased to decompress the contralateral central spinal canal and nerve root. The skillful removal of the PRSVB lies in carefully removing it bit-by-bit using the microscopic bone knife and taking it out using small straight forceps in time. The pathological changes of LSS with DLS are slow and complex until obvious symptoms appear. The nerve roots and dura mater remain compressed for a long time, and local inflammation may lead to scar adhesion. Therefore, it is necessary to distinguish the anatomical structure of the intervertebral space carefully during surgery before performing decompression operations. The adhesion between the dural sac, nerve root, and protrusion should be first loosened to reduce the risk of nerve and dural injury. Additionally, because osteoporosis patients are prone to bleeding and a blocked surgical field of vision, it is necessary to control bleeding strictly through radiofrequency coagulation and sufficient irrigation pressure. In patients with neurogenic claudication with unilateral or bilateral leg symptoms, the PRSVB compressing the traversing nerve roots and cauda equina must be removed to expand the central spinal canal, lateral recess, and foramen. Postoperative CT can show that the ventral and dorsal edges of the spinal canal are aligned and becoming smooth and continuous, which is similar to the postoperative findings of the reduction method in open surgery. We have created a model diagram to illustrate the key points further (Fig. 7).

In this study, the patients exhibited no significant progression of slippage during the last follow-up compared to that present preoperatively, indicating that PTED will not affect the natural process of LSS with DLS, which corroborates previous reports [14, 16, 20]. Ahuja et al. [27] found that the removal of more than 30% of the facet joint would increase spinal mobility and the pressure in the intervertebral disc. Foraminoplasty aims to remove the bone effectively from the ventral side of the SAP and protect the integrity of the facet joint. In this study, our technique only removed a small part of the facet joint, accounting for less than 10–20% of the total facet joint. Moreover, removing the PRSVB can avoid excessive removal of the back of the facet joint. The imaging results of this study showed that sufficient decompression and facet joint preservation were achieved in almost all patients. During the postoperative follow-up, no patients showed any further clinical or radiological segmental instability on dynamic radiographs; hence, this decompression technique may not produce obvious iatrogenic spinal instability.

Currently, there are many endoscopic decompression methods for LSS with DLS, such as percutaneous endoscopic interlaminar decompression [28], unilateral biportal endoscopic decompression [29], and microendoscopic decompression [30]. All these techniques adopt a posterior approach, providing surgeons with a friendly and familiar view and less tissue damage. The main concept of these methods is dorsal decompression, requiring general anesthesia and partial posterior joint resection. However, it is possible that the ventral PRSVB cannot be completely removed, the decompression of the contralateral nerve root is difficult, the decompression of foraminal stenosis is limited, and the surgical risk increases in elderly patients under general anesthesia. Additionally, elderly patients with LSS and DLS often have stenosis of the interlaminar space due to hyperplastic osteophytes and ossified LF; hence, posterior decompression to remove the dorsal bone and LF may be time-consuming. Excessive dorsal resection and decompression may lead to fractures and segmental instability [31], and the risk of dural injury increases.

The PTED technique used in this study is superior to the posterior approach in the degree of ventral decompression achieved. The PRSVB formed by DLS is an important pathological factor for CSS, lateral recess stenosis, and foraminal stenosis. PTED not only achieves 270° decompression of the nerve root but also removes the PRSVB with a good visual angle. When this part is removed, the area of the spinal canal is enlarged to relieve claudication [5]. Moreover, the enlargement of the ventral space ensures that the endoscope has enough space to pass in front of the dura mater to complete the decompression of the contralateral nerve root. Ultimately, the ideal surgical endpoint is identified by observing the entire circular fissure, epidural pulsation of the dural sac, and free movement of the nerve roots. Therefore, PTED combined with the removal of PRSVB has advantages in achieving decompression in patients with LSS and DLS. Notably, surgeons should evaluate the activity of the nerve root and decompress the ventral part of the dural sac repeatedly, including the epidural space and intervertebral disc. Although some patients with LSS and DLS experience unilateral lower limb symptoms, contralateral prophylactic decompression is still necessary when performing unilateral PTED. For patients with obvious symptoms in both lower limbs, the approach side should be determined based on imaging characteristics and patient signs, and bilateral PTED may have to be considered in some cases.

The average age of patients in this study was 69.5 years, and 84.1% (37/44) of them had comorbidities. The average age and incidence of preoperative comorbidities were higher in this study than in other endoscopic reports [26]; the VAS and ODI scores of the patients in this study improved significantly during follow-up. These excellent results are not only attributed to the removal of the PRSVB technique we introduced but also to the advantage of using local anesthesia in PTED. Additionally, the average operation time in this study was 67.8 min, which was shorter than that of open fusion surgery [32, 33]. Therefore, the use of local anesthesia provides surgical opportunities for elderly patients who refuse open fusion surgery or are weak, reduces the occurrence of complications related to fusion surgery and general anesthesia, and requires simple postoperative management. Local anesthesia can provide surgeons with nervous feedback; hence, the doctors can promptly cease activities that stimulate the nerve root to prevent further injury. From the point of view of population health, PTED is a treatment method with low medical costs and a relatively low risk of complications, which is beneficial to patients with DLS and LSS, who are the first choice for PTED treatment.

Endoscopic lumbar fusion surgery, as a reliable minimally invasive spinal technique, has been used to treat lumbar degenerative diseases [34]. Some scholars have reported that full-endoscopic trans-Kambin’s triangle lumbar interbody fusion was applied to the treatment of DLS with LSS, and achieved good results [35, 36]. This technique can not only remove the disc material under endoscope, but also achieve fixation or correction of spondylolisthesis through the implantation of pedicle screws and fusion cages. Therefore, for patients with potentially unstable or unstable DLS, fusion surgery should be given priority. However, some shortcomings of endoscopic fusion have also been reported, such as the lack of direct decompression of neural structure, residual postoperative pain and postoperative cage subsidence or migration [34]. Therefore, the choice of technology depends more on the pathological characteristics of the disease, patients’ symptoms and willingness, and cost-effectiveness. For stable DLS patients who only show symptoms related to nerve root compression, it is more reasonable and beneficial to choose PTED to relieve symptoms before fusion surgery is really needed.

In this study, there was one case of dural sac tear, which was caused by the bone knife accidentally slipping and tearing the dural sac because the surgical field of vision was seriously affected by profuse bleeding while removing the PRSVB in this osteoporosis patient. Therefore, the prerequisite for the next procedure is careful hemostasis to ensure clear vision. One patient had residue of herniation. During the revision operation, fragmented-free nucleus pulposus tissue was found at the nerve root canal, which may have been due to the lack of experience of orthopedic doctors in the early stages of the surgery, resulting in insufficient clearance of the intervertebral disc and migration of the nucleus pulposus. Therefore, repeated decompression of the epidural and intervertebral discs is necessary to prevent residual nucleus pulposus. One patient had postoperative dysesthesia, which could be attributed to the nerve root injury caused by repeated punctures and foraminoplasty [37].

This study has some limitations: Firstly, due to its strict inclusion criteria of this retrospective study, the sample size was relatively small, and the evaluation study of larger sample size should be carried out in the future. Secondly, although the short-term follow-up indicated that PTED with the removal of the PRSVB achieves good results, the long-term clinical effects and complications were unknown, and long-term follow-up was still needed. Thirdly, without a control group, the advantages of PTED combined with the resection of the PRSVB and single dorsal decompression cannot be compared and studied in detail. Fourthly, data such as the changes in the spinal canal area were not specifically assessed, as we attributed the relief of lower limb symptoms to the expansion of the vertebral canal. The relevant data will be considered in future research to illustrate the advantages of PTED with the removal of the PRSVB clearly.

## Conclusion

PTED combined with the removal of the PRSVB can significantly alleviate lower limb symptoms in patients with LSS with low-grade stable DLS, and the short-term efficacy is satisfactory. However, the long-term clinical effect needs to be further confirmed by continuous follow-up.

### Electronic supplementary material

Below is the link to the electronic supplementary material.

Additional file 1

## Data Availability

The data presented in this study are available on request from the corresponding author. The data are not publicly available due to privacy reasons.

## Abbreviations

CT: Computed tomography
CSS: Central spinal stenosis
DLS: Degenerative lumbar spondylolisthesis
LSS: Lumbar spinal stenosis
MRI: Magnetic resonance image
ODI: Oswestry Disability Index
LF: Ligamentum flavum
PTED: Percutaneous transforaminal endoscopic decompression
PRSVB: Posterosuperior region underneath the slipping vertebral body
SAP: Superior articular process
VAS: Visual analogue scale
